# Lysine-Specific Demethylase 1 in Breast Cancer Cells Contributes to the Production of Endogenous Formaldehyde in the Metastatic Bone Cancer Pain Model of Rats

**DOI:** 10.1371/journal.pone.0058957

**Published:** 2013-03-14

**Authors:** Jia Liu, Feng-Yu Liu, Zhi-Qian Tong, Zhi-Hua Li, Wen Chen, Wen-Hong Luo, Hui Li, Hong-Jun Luo, Yan Tang, Jun-Min Tang, Jie Cai, Fei-Fei Liao, You Wan

**Affiliations:** 1 Neuroscience Research Institute and Department of Neurobiology, Peking University, Beijing, People's Republic of China; 2 Institute of Biophysics, Chinese Academy of Sciences, Beijing, People's Republic of China; 3 Department of Anatomy, Basic Medical College, Zhengzhou University, Zhengzhou, People's Republic of China; 4 Central Laboratory, Shantou University Medical College, Shantou, People's Republic of China; 5 Department of Anatomy and Histology, School of Basic Medical Sciences, Peking University, Beijing, People's Republic of China; 6 Key Laboratory for Neuroscience, Ministry of Education/Ministry of Health, Beijing, People's Republic of China; Medical College of Georgia, United States of America

## Abstract

**Background:**

Bone cancer pain seriously affects the quality of life of cancer patients. Our previous study found that endogenous formaldehyde was produced by cancer cells metastasized into bone marrows and played an important role in bone cancer pain. However, the mechanism of production of this endogenous formaldehyde by metastatic cancer cells was unknown in bone cancer pain rats. Lysine-specific demethylase 1 (LSD1) is one of the major enzymes catalyzing the production of formaldehyde. The expression of LSD1 and the concentration of formaldehyde were up-regulated in many high-risk tumors.

**Objective:**

This study aimed to investigate whether LSD1 in metastasized MRMT-1 breast cancer cells in bone marrows participated in the production of endogenous formaldehyde in bone cancer pain rats.

**Methodology/Principal Findings:**

Concentration of the endogenous formaldehyde was measured by high performance liquid chromatography (HPLC). Endogenous formaldehyde dramatically increased in cultured MRMT-1 breast cancer cells *in vitro*, in bone marrows and sera of bone cancer pain rats, in tumor tissues and sera of MRMT-1 subcutaneous vaccination model rats *in vivo*. Formaldehyde at a concentration as low as the above measured (3 mM) induced pain behaviors in normal rats. The expression of LSD1 which mainly located in nuclei of cancer cells significantly increased in bone marrows of bone cancer pain rats from 14 d to 21 d after inoculation. Furthermore, inhibition of LSD1 decreased the production of formaldehyde in MRMT-1 cells *in vitro*. Intraperitoneal injection of LSD1 inhibitor pargyline from 3 d to 14 d after inoculation of MRMT-1 cancer cells reduced bone cancer pain behaviors.

**Conclusion:**

Our data in the present study, combing our previous report, suggested that in the endogenous formaldehyde-induced pain in bone cancer pain rats, LSD1 in metastasized cancer cells contributed to the production of the endogenous formaldehyde.

## Introduction

Bone metastasis is a common complication of cancer, and ∼70% of breast cancer patients have bone metastasis [1]. After metastasized into bone, cancer cells, as well as tumor associated immune cells, osteoblasts, osteoclasts secrete factors like nerve growth factor (NGF) [2], prostaglandin E2 (PGE2) [3] and endothelin [4]. Tumor metastasis into bone always leads to cancer pain [5], because nociceptors innervating bone are usually activated by tumor cells and the secreted factors which then produced bone cancer pain.

The level of formaldehyde was highly increased in the urine of patients with prostate and bladder cancers [6]. Our previous study reported that formaldehyde concentration increased in the bone marrow of breast MRMT-1 bone cancer pain rats and in tissues from breast cancer patients and lung cancer patients [7]. However, the mechanism of production of this endogenous formaldehyde by metastatic cancer cells was unknown in bone cancer pain rats.

Formaldehyde is ubiquitous in nature, and it is an endogenous chemical in most organisms including human [8]. Formaldehyde presents in all tissues, body fluids, and in blood with a concentration about 0.1 mM [9]. It is produced as a by-product from N-, O- and S-demethylation reactions in cells [8], and usually detoxified by L-glutathione (GSH) [10]. Enzymes in formaldehyde production mainly include histone demethylase 1 (LSD1) [11], serine hydroxymethyltransferase (SHMT) [12], dimethylglycine dehydrogenase (DMGDH) [13], sarcosine dehydrogenase (SDH) [13], sarcosine oxidase [14] and semicarbazide-sensitive amine oxidase (SSAO) [15].

LSD1 is an amine oxidase that removes mono- and di-methyl moieties from Lys4 of histone H3 and generates the demethylated H3 tail and formaldehyde [16], [17]. The expression level of LSD1 and concentration of formaldehyde were up-regulated in certain high-risk tumors, such as the prostate cancer [6], [11], [18], bladder carcinomas [6], [19], [20], lung cancer [7], [19], [21] and breast cancer [22].

In the present study, we proposed that LSD1 in cancer cells metastasized into bone marrow contributed to the production of endogenous formaldehyde in bone cancer pain rats.

## Materials and Methods

### Ethics statement

Female virgin Sprague-Dawley rats weighing 150–180 g were provided by the Department of Experimental Animal Sciences, Peking University Health Science Center. Animals were housed in cages with free access to food and water during experiment and were kept under natural diurnal cycle. All experiment processes were approved by the Animal Use and Care Committee of Peking university (Permit Number: LA2012-76) and in accordance with the guidelines of the International Association for the Study of Pain [23].

### Drugs preparation and application

Formaldehyde (USP grade, 37%, W/V, in water) solution, glutathione (GSH, a formaldehyde scavenger), and pargyline (an LSD1 inhibitor) were purchased from Sigma-Aldrich (St. Louis, MO, USA), and were dissolved in normal saline (NS). Drug doses were chosen according to other reports and to our preliminary data [24], [25]. Other reagents were analytical grade. In high performance liquid chromatography with fluorescence detection (Fluo-HPLC) experiments, all solvents were HPLC-grade.

Cultured MRMT-1 cells were exposed to pargyline (1 and 2 mM) for 30 h, and the concentration of formaldehyde in MRMT-1 cells was measured. In bone cancer pain model, the intraperitoneal injection of pargyline (0, 50, and 75 mg/kg) was given from day 3 to day 14 and then pain responses were examined.

### Cell culture

MRMT-1 rat mammary gland carcinoma cells (Novartis Oncology Research, Basel) were cultured in RPMI 1640 medium (Gibco). All cultures were kept in an incubator under a humid atmosphere (37°C, 95% air and 5% CO_2_).

### Establishment of rat model of bone cancer pain

A bone cancer pain model was established using SD female rats with MRMT-1 rat mammary gland carcinoma as in previous reports [7], [26]. After anesthesia, the left tibia of the rat was exposed and a 23-gauge needle was inserted into the intramedullary canal of the bone. Then it was removed and replaced with a long thin blunt needle attached to a 10-µl Hamilton syringe containing MRMT-1 cell suspension. A volume of 4 µl of suspension (containing 2×10^5^ cells) or phosphorylated buffer solution (PBS) was injected into the bone marrow cavity. The entry site on the bone was sealed with bone wax.

### Subcutaneous vaccination cancer model of MRMT-1 cells

This model was built to measure the formaldehyde concentration in solid MRMT-1 tumors. After ether anesthesia, rats were transplanted by subcutaneous injection of 10-µl cell suspension (containing 2×10^5^ MRMT-1 cancer cells) to the right side of the back [27]. Tumors and sera of these rats were taken out for the formaldehyde measurement.

### Formaldehyde measurement with Fluo-HPLC

Bone marrows, tumor tissues (subcutaneous vaccination sample), sera and cultured cancer cells were harvested for the formaldehyde measurement. As the bone cavity volume of rats was small and the amount of bone marrow tissue was little, 6 – 7 bone marrows were combined to one tube for HPLC measurement of formaldehyde. An HP 1100 HPLC instrument (Hewlett-Packard, UA) with fluorodetector was used. The method was described as in our previous reports [7], [28]–[31] and others [32]. The limit of detection of formaldehyde was 0.46 mg/ml (15.3 µM) [28]. According to the count, the quantity of MRMT-1 cells up to the limit of formaldehyde detection was 7.7 × 10^5^ cells.

### Low dose formaldehyde test for acute nociception

Rats were handled for 3 days before behavior experiments. After 20 min adaptation, the plantar of right hind paw was injected with formaldehyde (1 – 3 mM) at pH 6.0 (mimicking a moderate to severely acidic tumor microenvironment) with a 26-gauge needle. The length of time that the animals spent in flinching, licking, lifting or biting was recorded.

### Radiant heat test for thermal hyperalgesia in rats with cancer pain

Rats habituated to a plexiglas chamber on a transparent glass surface and allowed to acclimate for at least 20 min. At days 0, 7, 14 and 21 after inoculation of MRMT-1 cells into bone marrow, thermal hyperalgesia was tested with radiant heat apparatus (IITC Life Science Instruments). The radiant heat stimulus was a focused beam of light (4×6 mm in diameter) from a modified thermo-stimulator. The paw withdrawal latency in response to the radiant heat was recorded. Measurements were repeated for 5 times, and the mean value was calculated as the thermal threshold [33].

### Von Frey hair test for mechanical allodynia in rats with cancer pain

The animal was placed in a clear plexiglas compartment with a mesh floor and was habituated for 20 min. At days 0, 7, 14 and 21 after inoculation of MRMT-1 cells, mechanical allodynia was evaluated with *von* Frey hairs (Semmes-Weinstein Monofilaments, North Coast Medial Inc., San Jose, CA, USA). An ascending series of *von* Frey hairs (0.40, 0.60, 1.4, 2.0, 4.0, 6.0, 8.0 and 15.0 g) were applied perpendicular to the mid-plantar surface for 6–8 s of each hind paw. A trial began with the application of the 2.0 g *von* Frey hair filament. A positive response was defined as a withdrawal of hind paw upon the stimulus. When a positive response to a stimulus occurred, the next lower *von* Frey hair was applied, otherwise when a negative response occurred, the next higher hair was applied. The testing contained more than 5 stimuli after the first change in response occurred, and the pattern of response was converted to a 50% paw withdrawl threshold (PWT) using the method as previously reported [7],[34]–[42].

### Cell viability assay

To examine the inhibition of pargyline (an LSD1 inhibitor) on cell growth, cell viability assay was determined using WST-8 dye (Beyotime Inst Biotech, China). Cells were diluted in RPMI 1640 medium and seeded in 96-well plates. After overnight adherence, MRMT-1 cells (2×10^5^ cells/ml) were treated with different concentrations of pargyline (1, 2, 5, 10 and 20 mM), then added 10 µl WST-8 dye to 100 µl medium for 1 h at 37°C. Optical density (OD) values were determined at 450 nm in a microplate reader (Bio-Rad Laboratories, Hercules, CA, USA).

### Western blotting for the detection of protein expressions of LSD1, monomethyl-H3K4, demethyl-H3K4 and H3

Cells and bone marrows were prepared by using lysis buffer (containing 50 mM Tris-HCl pH 8.0, 150 mM NaCl, 1 mM EDTA, 1% NP40, 0.5% deoxycholic acid, 0.1% SDS, 1 mM Na_3_VO_4_, 1 mM phenylmethylsulfonyl fluoride) for 1 h at 4°C followed by centrifugation. Proteins were determined on SDS-PAGE gels and transferred onto PVDF membranes [37], [41]. Primary antibodies were used including against LSD1 (1∶1000, Cell Signaling, Boston, US), monomethyl-H3K4 (1∶1000, Abcam, Cambridge, UK), demethyl-H3K4 (1∶1000, Abcam, Cambridge, UK) and H3 (1∶10000, Abcam, Cambridge, UK).

### Immunofluorescent detection of tumor cells in the bone marrow

This part aimed to distinguish the tumor cells from the bone marrow cells. The rat bone marrow was rushed out by RPMI-1640 medium and then separated by density centrifugation using Ficoll (density 1077 g/ml, Biochrom, Berlin, Germany). Cells (1×10^4^/ml) were spun down onto a glass slide. The slides were dried with cold air, and fixed with acetone at 4 °C for 10 min. After incubation with the LSD1 antibody (1∶100, Cell Signaling, Boston, US) for 24 h at 4 °C, the slides were incubated with secondary antibody labeled with Alexa Fluo 568 (1∶1000, Invitrogen, Mount Waverly, VIC, Australia) for 1 h at room temperature. Pan-cytokeratin has been widely used as a specific marker in identification and classification of epethelium-derived tumors like MRMT-1 cells. Then the slides were incubated with pan-cytokeratin antibody labeled with FITC (1∶50, Abcam, Cambridge, UK) and Hoechst (1∶10000, Invitrogen, Mount Waverly, VIC, Australia) for 2 h at room temperature. Slides were analyzed for the presence of tumor cells using laser scanning confocal microscope (SP5 Leica, Wetzlar, Germany).

### Statistical analysis

Data were expressed as mean ± standard error of the mean. Data were analyzed by *t*-test, one- or two-way analysis of variance (ANOVA) followed by Dunnett's multiple comparison test where appropriated. Statistical significance in all cases was set as *p*<0.05.

## Results

### Formaldehyde concentration increased in cultured MRMT-1 cells in vitro and in tumor tissues in vivo

**Figure 1:**
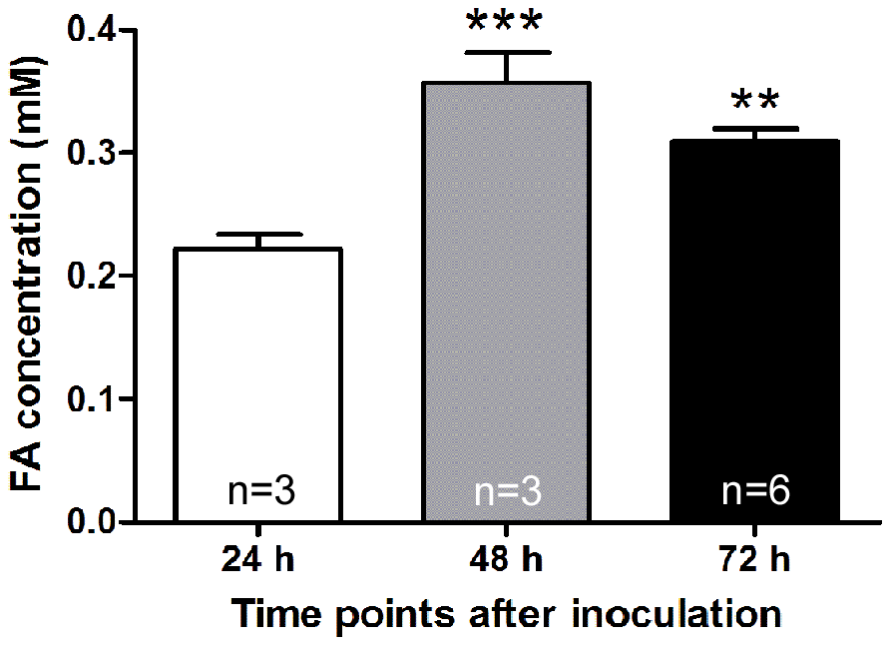
Formaldehyde (FA) concentration in the cultured MRMT-1 cancer cell line. ** *p*<0.01, *** *p*<0.001, compared with the 24 h group.

**Figure 2:**
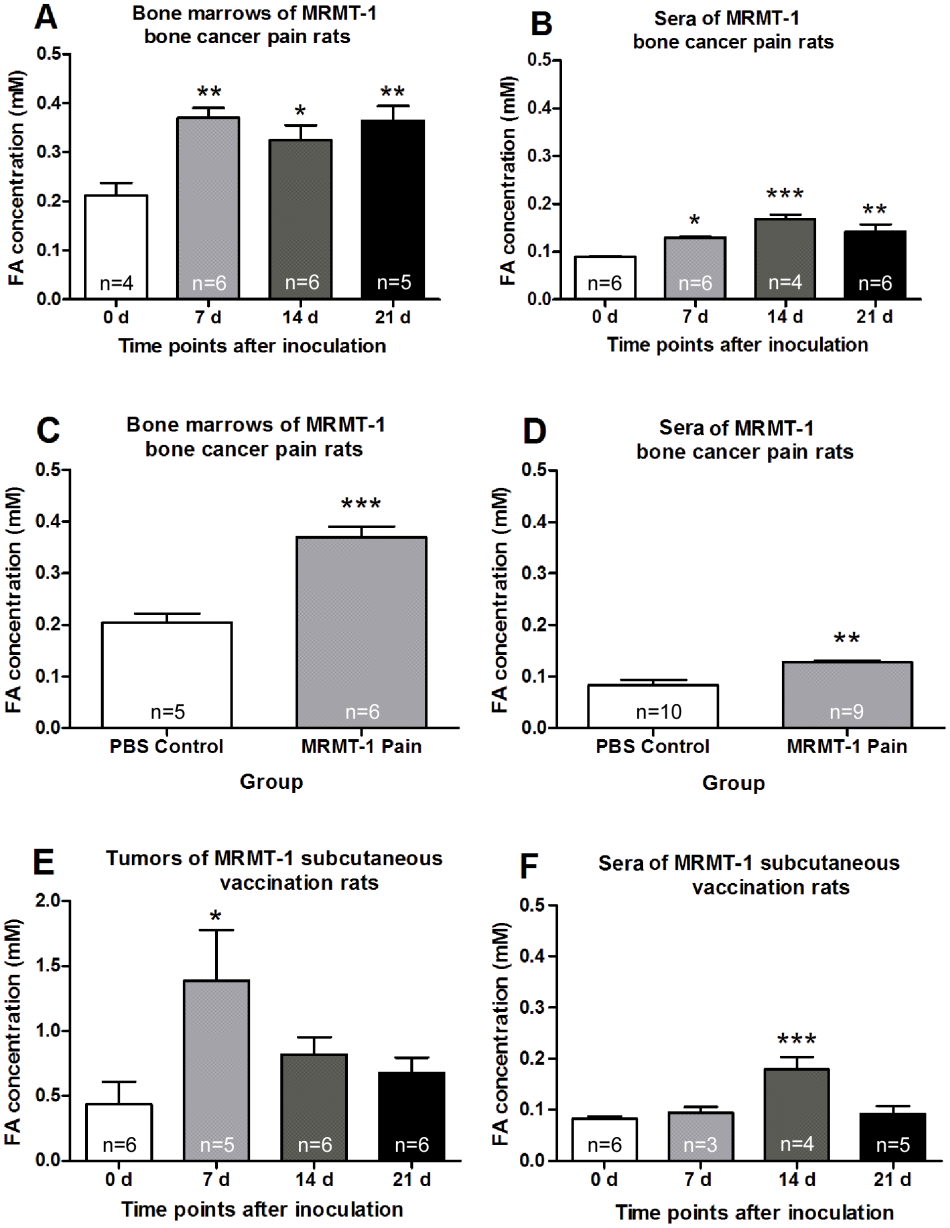
Measurement of formaldehyde concentration in different tissues. (A) and (B) Bone marrows of the tibia bone and sera at different times after MRMT-1 inoculation in cancer pain rats. (C) and (D) Bone marrows of the tibia bone and sera at day 7 in MRMT-1 cancer pain rats or PBS control rats. (E) and (F) Tumors and sera at different times after MRMT-1 subcutaneous vaccination in rats. * *p*<0.05, ** *p*<0.01, *** *p*<0.001, compared with day 0 group or PBS 7 d group.

Formaldehyde concentration in the cultured tumor cell line was measured. Formaldehyde concentration in cultured rat breast MRMT-1 cells increased significantly at 48 h and 72 h compared with that 24 h after inoculation (Fig. 1). Compared with that at day 0, the formaldehyde concentration increased significantly in bone marrows and sera of rats with bone cancer pain at days 7, 14 and 21 (Fig. 2, A and B). Compared with PBS group, formaldehyde concentration in bone marrows and sera also increased significantly in bone cancer pain group at day 7 after inoculation (Fig. 2, C and D). Compared with that at day 0, formaldehyde concentration increased significantly in tumors of the MRMT-1 subcutaneous vaccination model 7 days after inoculation (Fig. 2E), and in sera at day 14 after inoculation (Fig. 2F).

### Formaldehyde at low concentration induced acute pain behaviors

**Figure 3:**
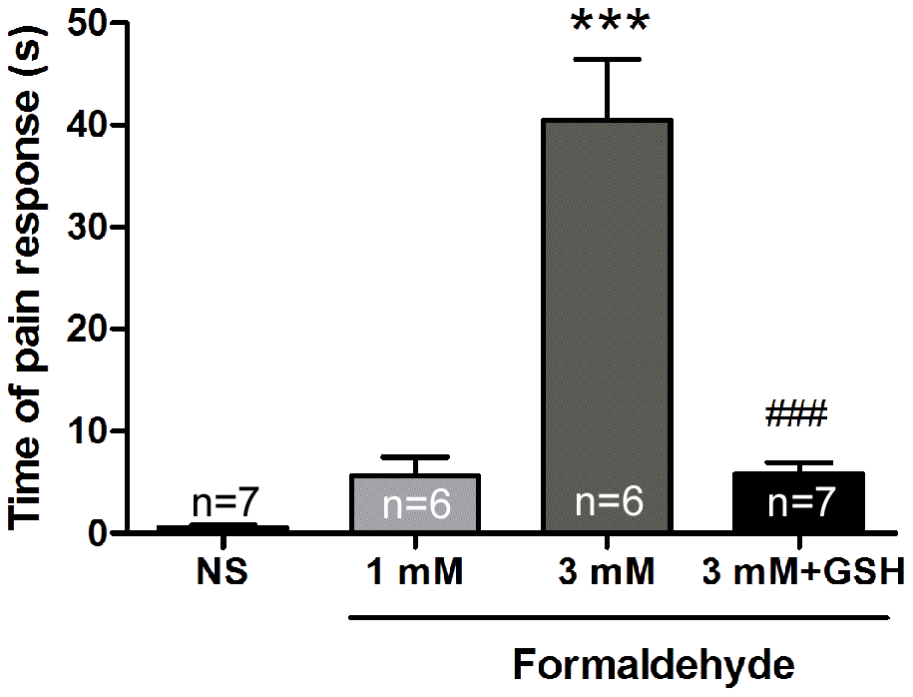
Inhibition of glutathione (GSH, a formaldehyde scavenger) on formaldehyde-induced acute pain responses. *** *p*<0.001, compared with the NS group; ^###^ *p*<0.001, compared with the 3 mM formaldehyde group.

It was well known that as in classical formalin test, high concentration of formaldehyde (5% formalin, 1667 mM) as a chemical irritant induced inflammatory pain [43], [44]. However, in the present and our previous studies, formaldehyde concentration in bone marrows of bone cancer pain models and solid tumors of cancer patients was relatively very low (≤ 3 mM) [7]. Therefore we would like to know whether formaldehyde at such a pathologically low concentration could induce pain responses. Intraplantar injection of formaldehyde at doses of 1 and 3 mM to the right hind paw, time of pain behaviors within 5 min was recorded in the injected paw of normal rats. Compared with the NS group, 3 mM formaldehyde increased the pain response time. Pre-application GSH (a formaldehyde scavenger) decreased the formaldehyde-induced pain responses (Fig. 3). These data suggested that formaldehyde at a pathological concentration as low as in the cancer tissues could induce pain behavior.

### LSD1 protein expression in cancer cells and tissues

**Figure 4:**
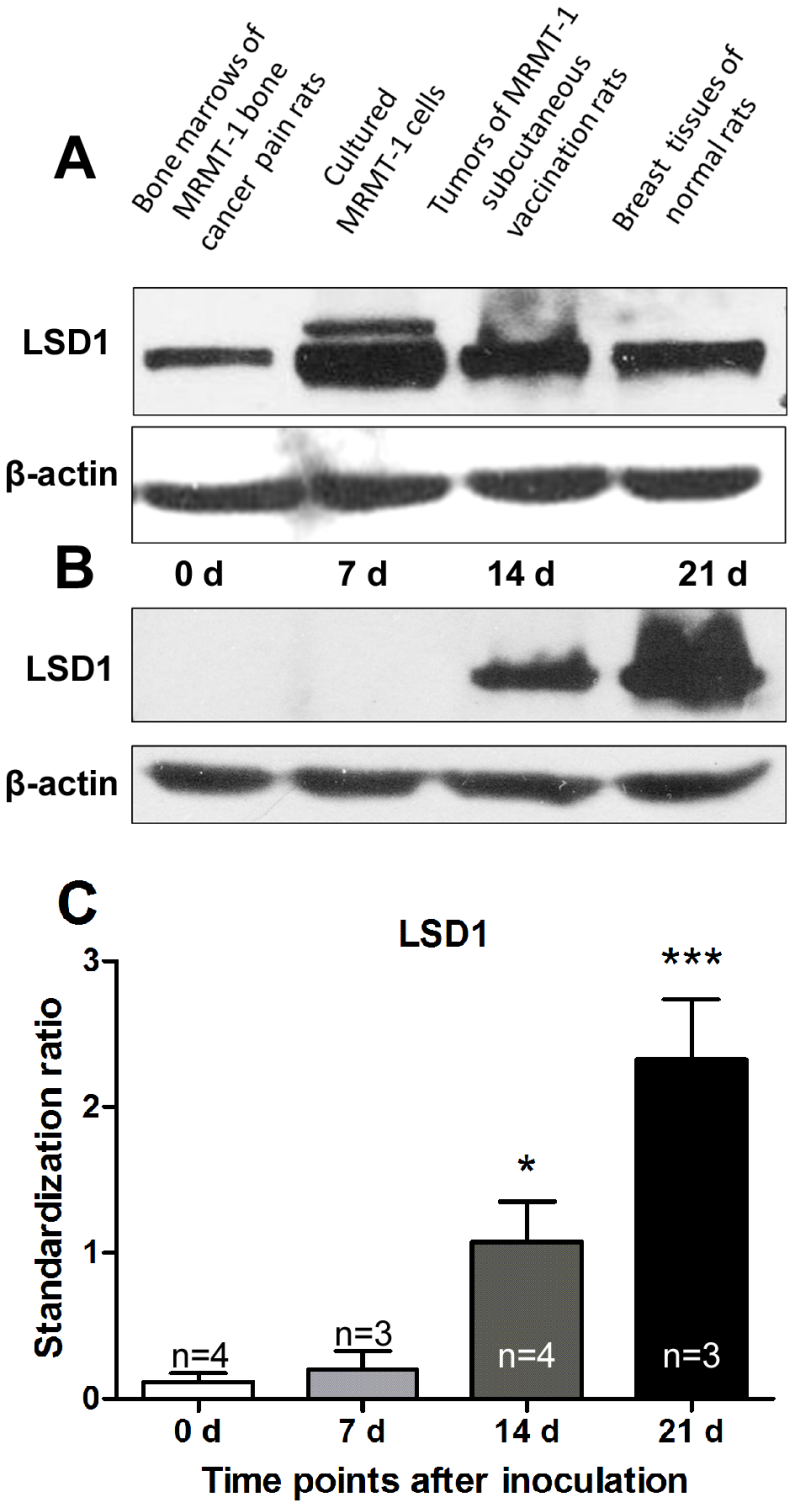
LSD1 expression with Western blotting detection. (A) In tibia bone marrows of bone cancer pain rats at day 14 after MRMT-1 inoculation, MRMT-1 cells cultured for 48 h, MRMT-1 subcutaneous tumors of rats at day 14 after vaccination and breast tissues of normal rats. (B) In tibia bone marrows of bone cancer pain model of rats at days 0, 7, 14 and 21 after MRMT-1 inoculation. (C) Statistical analysis of results in Figure 4B. * *p*<0.05, *** *p*<0.001, compared with day 0 (0 d) group.

Expression of LSD1 was detected by Western blotting analysis in bone marrows of MRMT-1 bone cancer pain rats, cultured MRMT-1 cells, subcutaneous vaccination tumors of MRMT-1 in rats and breast tissues of normal rats. LSD1 expression was found in these samples (Fig. 4A). Furthermore, expression of LSD1 in the bone marrow was analyzed in MRMT-1 bone cancer pain model. Compared with that at day 0, the level of LSD1 was significantly increased at days 14 and 21 after MRMT-1 inoculation (Fig. 4, B and C).

**Figure 5:**
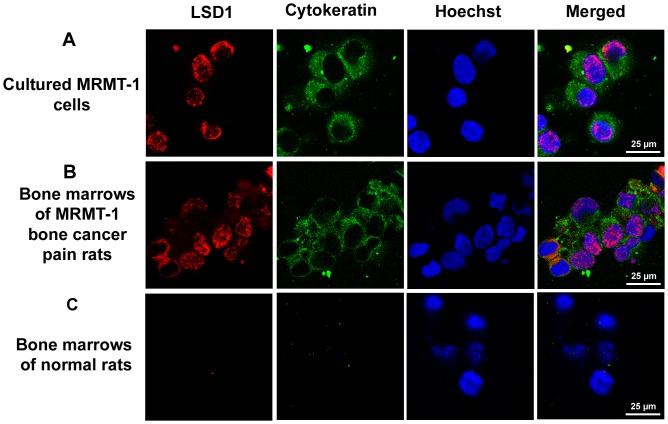
Immunofluorescent labeling of LSD1 (red) with cytokeratin (green) and Hoechst (blue). (A) In cultured MRMT-1 rat breast cancer cells. (B) In tibia bone marrows of MRMT-1 bone cancer pain rats. (C) In bone marrows of normal rats. Bar  = 25 µm.

The location of LSD1 was shown by immunofluorescence in cultured rat breast cancer cell line MRMT-1 cells, in bone marrows of MRMT-1 bone cancer pain rats and in bone marrows of normal rats. Pan-cytokeratin is a marker for epithelium-derived tumor like MRMT-1 breast cancer cells. It was found that LSD1 expression mainly located in nuclei of cytokeratin-positive MRMT-1 cells (cultured MRMT-1 tumor cells and bone marrow cells of MRMT-1 bone cancer pain rats), not in bone marrow cells of normal rats (Fig. 5). Few LSD1 located in the cytoplasms of bone marrow cells of MRMT-1 bone cancer pain rats (Fig. 5B). These data suggested that LSD1 mainly expressed in the nucleus of the MRMT-1 cancer cell after inoculation into bone marrow *in vivo* as well as in the cultured one *in vitro*.

### Inhibition of LSD1 function decreased production of formaldehyde in cultured MRMT-1 cells

**Figure 6:**
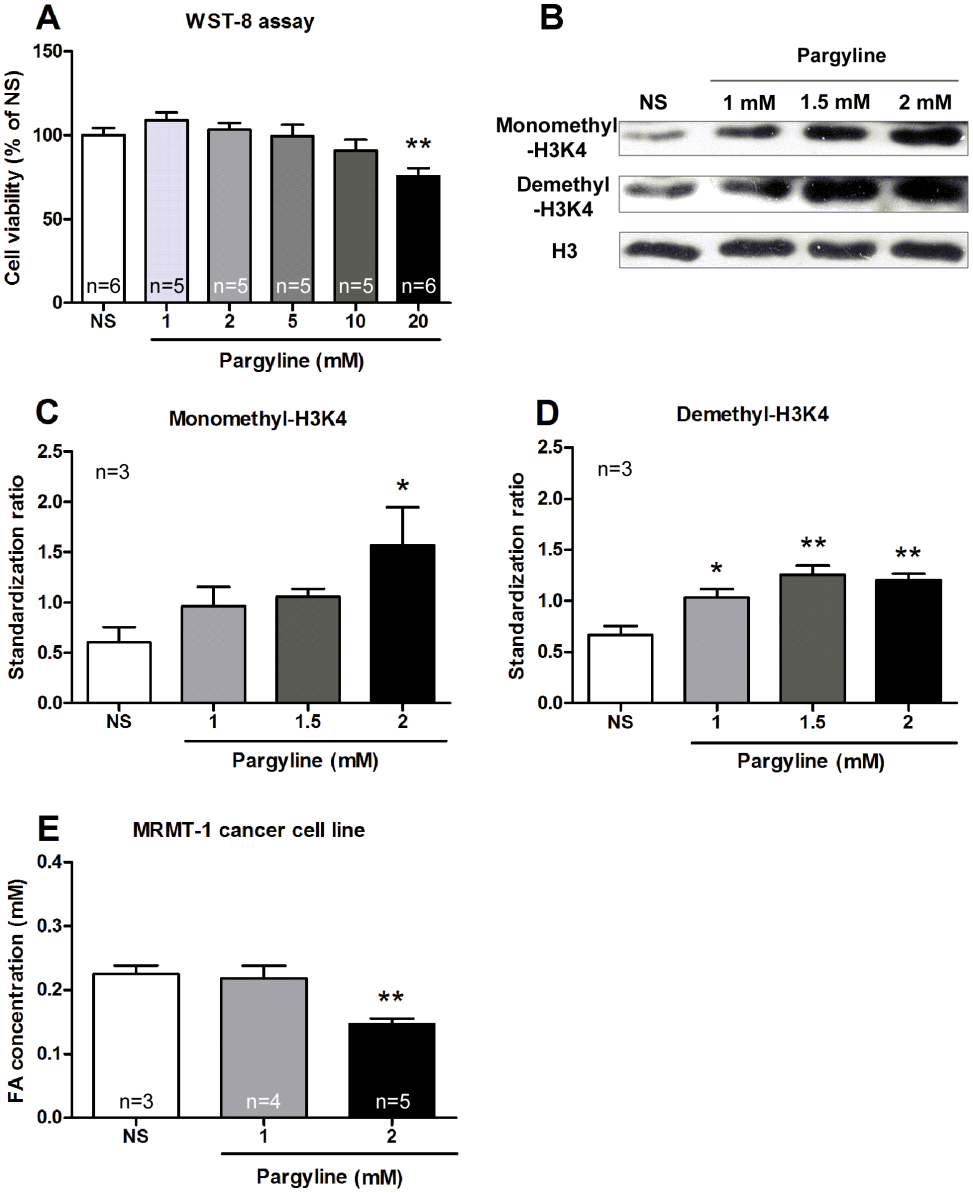
Inhibition of pargyline on LSD1 expression and the subsequent production of formaldehyde in MRMT-1 cells. (A) WST-8 assay for cell viability of MRMT-1 cells after treatment with pargyline (1–20 mM) for 30 h. (B–D) The expression of monomethyl-H3K4 and demethyl-H3K4 in cultured MRMT-1 cells after treatment with pargyline (1, 1.5 and 2 mM) for 30 h. H3 was used as a loading control. (E) Formaldehyde concentration of MRMT-1 cancer cells after treatment with pargyline (1 and 2 mM). * *p*<0.05, ** *p*<0.01, compared with the NS group.

To probe the role of LSD1 in regulation of production of formaldehyde, cultured MRMT-1 cells were treated with an LSD1 inhibitor (pargyline). The cell viability assay results showed that pargyline at 1–10 mM had no effect, while at 20 mM inhibited the cell growth (Fig. 6A), so we chosed pargyline at 1–2 mM for further experiments. Exposure of MRMT-1 cells to pargyline (1, 1.5 or 2 mM) for 30 h significantly increased monomethyl-H3K4 and demethyl-H3K4 (Fig. 6, B–D). These results indicated that pargyline inhibited the LSD1 function in cultured MRMT-1 cells.

After cultured MRMT-1 cells were exposed to pargyline (1 and 2 mM) for 30 h, the concentration of formaldehyde in collected MRMT-1 cells was measured. Pargyline at 2 mM decreased the concentration of formaldehyde in MRMT-1 cells, while pargyline at 1 mM had no effect (Fig. 6E). These results indicated that LSD1 in MRMT-1 cells participated in the production of endogenous formaldehyde.

### LSD1 inhibitor attenuated bone cancer pain behaviors in rats

**Figure 7:**
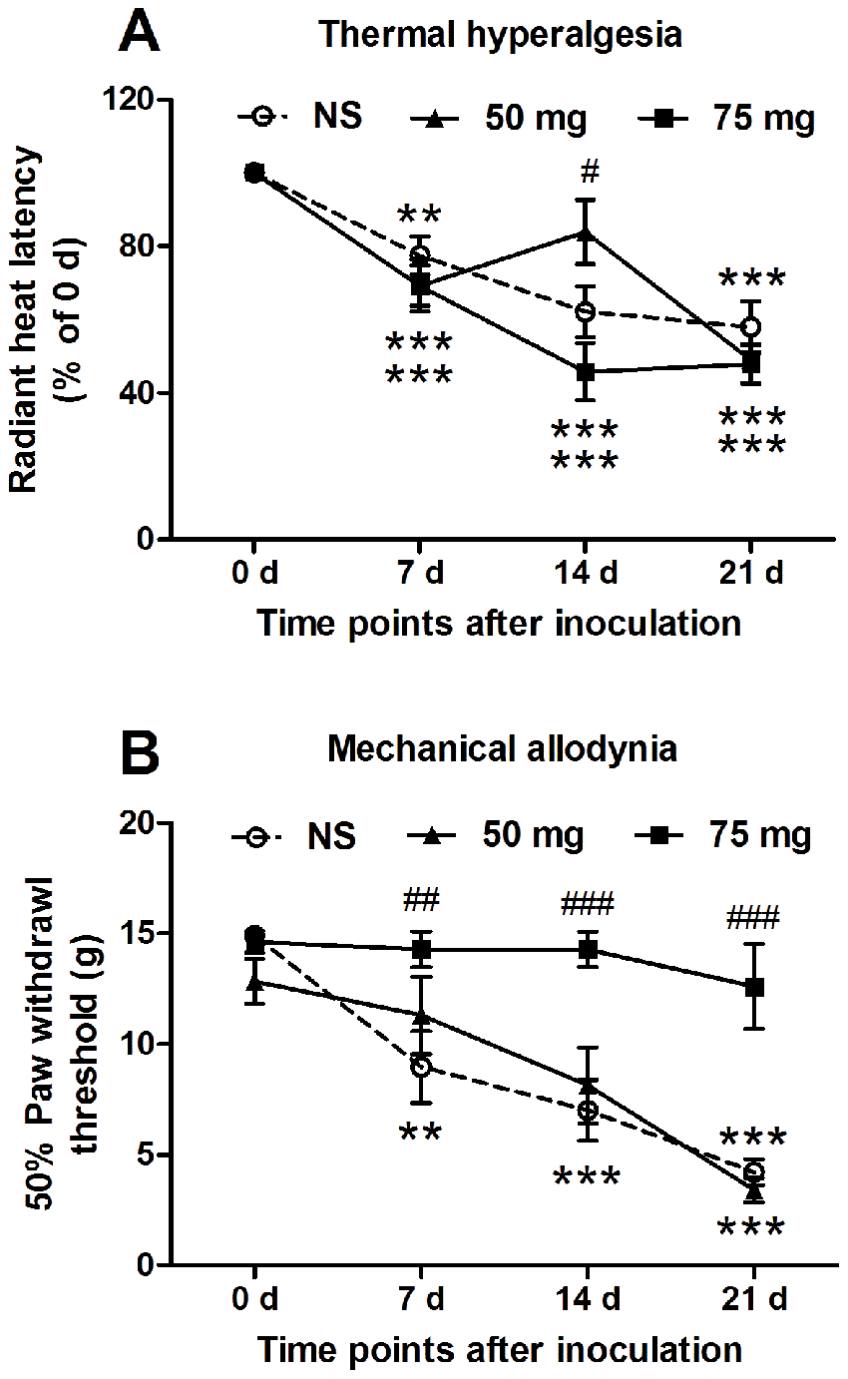
Inhibition of pargyline on bone cancer pain behaviors in rats. (A) Thermal hyperalgesia. (B) Mechanical allodynia. ** *p*<0.01, *** *p*<0.001 compared with that at day 0 (0 d); ^#^ *p*<0.05, ^##^ *p*<0.01, ^###^ *p*<0.001, compared with the NS group. *n* = 6–11.

As shown in Fig. 7, pargyline (an LSD1 inhibitor) was intraperitoneally injected from day 3 to day 14 after inoculation of MRMT-1 cancer cells to establish bone cancer pain model of rats. Pain behaviors including thermal hyperalgesia and mechanical allodynia were observed at days 0, 7, 14 and 21 after inoculation of MRMT-1 cancer cells into bone marrow. It was found that thermal hyperalgesia was attenuated by pargyline (50 mg/kg) at day 14 after MRMT-1 inoculation (Fig. 7A), and mechanical allodynia was inhibited by pargyline (75 mg/kg) at days 7, 14 and 21 after MRMT-1 inoculation (Fig. 7B).

## Discussion

### Cancer cell line and tumor tissues produced endogenous formaldehyde to a pathological concentration to induce pain

Formaldehyde exists in almost all normal tissues and body fluids. For example, its physiological concentration in human blood is about 0.1 mM [7], [9], [45]. In the urine of patients with bladder or prostate cancer, formaldehyde concentration was significantly elevated (2–8 folds) [6]. Formaldehyde concentration was also increased in the expiration of breast cancer patients and in lymphocytes in chronic lymphocytic leukemia [46], [47]. Our previous study reported that formaldehyde concentration increased in cultured tumor cell lines, in bone marrows of MRMT-1 bone cancer pain model rats and in tissues from breast cancer and lung cancer patients [7]. In the present study, we confirmed the production and elevated concentration of formaldehyde in cultured MRMT-1 cells *in vitro* and in rat tumor tissues *in vivo*. In MRMT-1 cell lines, formaldehyde concentration was increased (∼2 folds) (Fig. 1). Furthermore, we found that formaldehyde concentration elevated (∼2 folds) in both bone marrows and sera at days 7, 14 and 21 after MRMT-1 inoculation into tibia in bone cancer pain rats (Fig. 2, A – D). In the MRMT-1 subcutaneous vaccination model of rats, formaldehyde concentration was also elevated (∼2 folds) in tumors and sera (Fig. 2, E and F). These results indicated that the formaldehyde concentration was elevated in cultured breast cancer cell lines *in vitro*, in bone marrows and sera of MRMT-1 bone cancer pain model rats, in tumors and sera of MRMT-1 subcutaneous vaccination model rats *in vivo*. Combined with our previous study, the present study showed that the pathological concentration of formaldehyde in tumor tissues ranged from 0.3 to 3.0 mM in both rats and patients suffering from cancer pain.

More interestingly, we would like to know whether formaldehyde at such a range of low pathological concentrations could induce pain responses. We all know that in the classical formalin test, the formaldehyde concentration is 5%, i.e. 1667 mM. It was also reported that formaldehyde at a concentration 0.1% –2.5% (33–834 mM) could also induce acute pain responses [48], [49]. Does formaldehyde induce pain at a low concentration (≤3 mM, pH = 6.0) which is much lower than that in the classical formalin test? In normal rats, our results showed that intraplantar injection 3 mM formaldehyde induced acute pain responses, GSH (an endogenous formaldehyde scavenger) could inhibit this formaldehyde-induced pain responses (Fig. 3). Furthermore, formaldehyde (1 mM) under an acidic environment (pH  = 5.0) elicited C-fiber discharges *in vivo* in our previous study [7]. These results indicated that formaldehyde at a concentration as low as that in the rat tibia bone marrow after inoculation of MRMT-1 cancer cells into tibia could induce pain behavioral responses.

In MRMT-1 bone cancer pain rats, after bone destruction, nerve fiber endings in bone were exposed to the elevated formaldehyde. Besides, osteoclastic activity and tumors metabolism produced an acidic microenvironment in the bone marrow [50], [51]. In this situation, endogenous formaldehyde (although its concentration was ≤3 mM) from cancer tissues could induce metastatic bone cancer pain through activation of ion channels in nerve fibers especially under tumor acidic environment, for example TRPV1 [7].

### LSD1 expression increased in MRMT-1 cells metastasized into bone marrow

From our results and the above discussion, we can see that endogenous formaldehyde produced by cancer tissues might play an important role in bone cancer pain. In the present study, we would like to ask a further interesting question what the enzyme is in the MRMT-1 cells metastasized into bone marrow to produce the endogenous formaldehyde. As noted in the introduction part, endogenous formaldehyde can be mainly produced by catalytic enzymes like LSD1 [11], [52], SHMT [12], [53] and DMGDH [13], [54], and it is detoxified by GSH [10]. LSD1 took an important part in chromatin remodeling and transcriptional regulation in breast cancer cells [22] including MRMT-1 cancer cell line as well as in many other cancers such as the prostate cancer [6], [11], [18], the bladder carcinomas [6], [19], [20] and the lung cancer [7], [19], [21]. So, as a continuation of our previous study, here we focused on LSD1 in MRMT-1 breast cancer cells metastasized into bone marrows in the production of the endogenous formaldehyde in bone cancer pain rats.

Our results showed that LSD1 expressed in cultured MRMT-1 cells and bone marrows of MRMT-1 bone cancer pain rats (Fig. 4A). It mainly located in nuclei of cancer cells either in cultured MRMT-1 cells *in vitro* or in those MRMT-1 cells inoculated into bone marrows *in vivo* (Fig. 5). The level of LSD1 increased significantly at days 14 and 21 in bone cancer pain rats (Fig. 4, B and C). The formaldehyde concentration elevated (∼2 folds) in both bone marrows and sera at days 7, 14 and 21 in bone cancer pain rats. So, in bone cancer pain rats, LSD1 expression increased in inoculated MRMT-1 cancer cells.

### Inhibition of LSD1 decreased formaldehyde concentration and bone cancer pain

We would like to know whether this increased LSD1 in inoculated MRMT-1 cancer cells mediated the endogenous formaldehyde production to induce subsequent cancer pain. The MRMT-1 cell viability assay results showed that pargyline (an inhibitor of LSD1) at 1–10 mM had no effect on the cell growth.

We analyzed the effects of pargyline on formaldehyde concentration *in vitro.* Pargyline (2 mM) increased the protein expression of monomethyl-H3K4 and demethyl-H3K4, but did not influence the cell viability in cultured MRMT cells. These results suggested that pargyline inhibited the LSD1 function *in vitro* at the dose without any influence on the cell viability. The treatment of pargyline (2 mM) also decreased formaldehyde concentration in cultured MRMT-1 cells, suggesting that this increased LSD1 expression contributed mainly to the formaldehyde production in MRMT-1 cells. Limited by the technical immaturity in the assay of LSD1 activities in the tissues, we did not analyze the LSD1 enzyme activity in the present study.

Furthermore, we analyzed effects of pargyline on bone cancer pain *in vivo*. In the bone cancer pain model of rats, thermal hyperalgesia was attenuated by intraperitoneal injection of pargyline at day 14 after MRMT-1 inoculation, and mechanical allodynia was inhibited by pargyline at days 7, 14 and 21 (Fig. 7). These results *in vitro* and *in vivo* suggest that the inhibition of LSD1 could decrease the formaldehyde production, and then inhibit the subsequent bone cancer pain. By the way, we noted that thermal hyperalgesia and mechanical allodynia were attenuated by different concentrations of pargyline (50 or 70 mg/kg). This phenomenon maybe because formaldehyde had differential effects in different pain modalities (like thermal and mechanical) [55], and needs further investigation.

In the cancer research field, LSD1 was usually taken as a predictive marker for aggressiveness of cancer cells. It promoted cell phase transition, proliferation, migration and invasion [21], [22], [56]–[58]. LSD1 expression was highly upregulated in the poorly differentiated neuroblastoma, and strongly associated with adverse clinical outcome and inversely correlated with differentiation [59]. In the present study, we focused on the role of LSD1 on the production of endogenous formaldehyde and on cancer pain behaviors in bone cancer pain rats. We proposed that LSD1 might also provide a marker and a novel therapeutic target for bone cancer pain. Even though there was a limitation in the study that pargyline may inhibit growth of MRMT-1 cells in bone marrow of cancer pain rats and decrease tumor-secreted factors, it is still reasonable to believe that LSD1 in cancer cells metastasized into bone marrow contributed to the production of endogenous formaldehyde and cancer pain behavior in bone cancer pain rats.

LSD1 increases in many types of cancers, however, mechanisms of LSD1 increase remain an interesting question for cancer research as well as cancer pain research. In addition to LSD1, there were some other catalytic enzymes possibly involved in the production of endogenous formaldehyde, for example SHMT and DMGDH. On the other hand, there were formaldehyde degrading enzymes in the tissue, such as class I alcohol dehydrogenase (ADH1), aldehyde dehydrogenase 2 (ALDH2) and class III alcohol dehydrogenase (ADH3) [60], [61]. These catalytic and degrading enzymes of formaldehyde were considered to have critical roles in many cancer pathogenesis, such as breast and lung cancers [53], [62]–[64]. The possible role of these catalytic and degrading enzymes in formaldehyde production and cancer pain is of interest in further investigation.

In conclusion, our data in the present study, combining our previous report, suggested that in the endogenous formaldehyde-induced pain in bone cancer pain model of rats, LSD1 in metastasized cancer cells contributed to the production of the endogenous formaldehyde.
